# Position-Related Differences in Cardiorespiratory Functional Capacity of Elite Basketball Players

**DOI:** 10.2478/v10078-011-0082-1

**Published:** 2011-12-25

**Authors:** Audrius Gocentas, Nijole Jascaniniene, Stanislaw Poprzęcki, Jan Jaszczanin, Algirdas Juozulynas

**Affiliations:** 1 State Research Institute Centre for Innovative Medicine Žygimantų 9, LT-01102 Vilnius; 2 Vilnius Pedagogical University Studentu 39, LT- 08106, Vilnius; 3 The Jerzy Kukuczka Academy of Physical Education in Katowice; 4 University of Szczecin, Al. Piastow 40B, blok 6, 71-065 Szczecin, Poland

**Keywords:** Oxygen uptake, basketball, aerobic power, perimeter & post players

## Abstract

The purpose of this study was to examine possible differences in cardio respiratory functional capacity between perimeter and post elite basketball players. The subjects included 42 highly trained basketball players subdivided into groups of perimeter and post players. Point guards, shooting guards and small forwards were involved in the group of perimeter players, while power forwards and centers represented the group of post players. All players performed a standardized exercise test to evaluate maximal oxygen uptake using a cycle ergometer and automated breath-by-breath system VMAX229C. Collected data of power, heart rate, pulmonary ventilation and gas exchange were compared between the groups of perimeter and post players. Significant differences in anthropometric features between the investigated subgroups were observed. Post players were heavier and taller. Therefore, the perimeter players had significantly higher values of VO_2max_ and relative power. VO_2max_ was related to relative power. Relations between those variables can be described by linear regression. Given regressions can be used as a source of typical values for male basketball players. The results indicate that the empirical repartition of basketball players into perimeter and post players has not only a morphological but a physiological basis as well.

## Introduction

According to the American College of Sports Medicine (ACSM), the purpose of an exercise test is to determine the responses of the subject to efforts at given levels, and from this information to rate probable performance in life or professional activities. Despite its limited utility in asymptomatic subjects, cardiopulmonary exercise testing is part of the health assessments of elite athletes (Lauer et al., 2005; Arena et al., 2007). Basketball is a sport discipline with a significant distribution of participants according to a playing position on the court (Hughes et al., 2002; Ziv et al., 2009). Generally basketball players are divided into five categories regarding playing position. There are point guards, shooting guards, small forwards, power forwards, and centers. The traditional subdivision into five categories is very provisory because of the ability of the players to contest in several playing positions. On the other hand, very often drills are subdivided into perimeter and post players during modern basketball practices. There is a growing recognition of all guards and small forwards as perimeter players, while power forwards and centers as post players. This practical distribution is in accordance with criteria of performance for players of different positions (Trninic et al., 2000).

Training-related phenotype changes in athletes depend mainly on the intensity, frequency and duration of exercise as well on environmental conditions (Vamvakoudis et al., 2007; Manzi et al., 2010). If perimeter and post players have different drills and goals during practices, it is reasonable to expect from them a different functional capacity. Consequently typical values of power, oxygen uptake, ventilation and heart rate could be observed using standardized exercise tests for perimeter and post players. The typical values of main cardiorespiratory indices in basketball were described, analysed and compared in respect to age, gender, level of performance, modification of rules and other external factors (Apostolidis et al., 2004; Laplaud et al., 2004; Gocentas et al., 2005; Sallet et al., 2005; Cormery et al., 2008; Castagna et al., 2009; Metaxas et al., 2009; Narazaki et al., 2009; Ziv et al., 2009). Despite the increased popularity of basketball a number of physiological investigations of the players are inadequate.

The aim of this study was to compare the physical capacity of elite basketball players in terms of practical distribution into perimeter and post players and to determine the differences in variables that determine functional capacity such as oxygen uptake and anaerobic threshold.

## Material and methods

### Subjects

Forty-two healthy professional male basketball players were enrolled in this investigation. The players were differentiated into perimeter and post groups based on the decisions of the coaching staff. Written informed consent for participation in this study was obtained from all athletes in accordance with the code of ethics. Moreover, the study protocol was approved by the local Ethics Committee. The cardiorespiratory exercise testing was part of a biomedical examination required before players signed or extended their contracts with the team. Body height (to the nearest cm) and body mass (to the nearest 0.1 kg) were recorded with subjects barefoot and otherwise dressed in exercise clothing, and used to calculate the body mass index (BMI).

### Exercise Testing Protocol

Each subject was well rested before the test, and had not performed hard physical activity during the preceding 24 hours. All tests were carried out under laboratory conditions that complied with the regulations of the American Thoracic Society (ATS, 2003). Athletes underwent the exercise test on an electronically braked cycle ergometer (ERGOMETRICS 800; Ergoline, Bitz, Germany). Power output was increased by from 25 to 30 Watts (W) every minute, and the pedaling cadence was kept constant at 60–70 revolutions per minute (rpm). The test was terminated when the athlete evidenced one of the following: a respiratory exchange ratio equal to or greater than 1.10, a heart rate plateau despite the continued increase in workload, or an oxygen uptake plateau despite the continued increase in workload (Wasserman et al., 1999). Exercise testing was terminated prematurely if the athlete experienced leg discomfort that prevented him from pedaling effectively. Gas exchange data were collected continuously using the automated breath-by-breath system (VMAX229C; Sensormedics Corp., Yorba Linda, CA, USA). The flow/volume sensor was calibrated immediately prior to each test by manually pumping a 3-liter syringe through the flow-meter at a rate similar to that achieved during the exercise test. A 12-lead electrocardiogram, heart rate, and blood pressure were recorded during exercise and for 10 minutes of recovery post-exercise. Samples were taken every 20 seconds for evaluation of lung ventilation, heart activity and oxygen uptake indicators. The anaerobic threshold was identified by conventional criteria using the V-slope method (Wasserman et al., 1999). In all investigated subjects, the following variables were evaluated: oxygen uptake expressed at rest per kg of subject’s body mass (VO_2rest_), oxygen uptake at the anaerobic threshold (VO_2AT_), oxygen uptake at the peak of exercise (VO_2max_), work rate at the anaerobic threshold (W_AT_), work rate at the peak of exercise (W_max_), relative work rate expressed as the peak of work rate per kg of subject’s body mass (W_relat_), heart rate at the anaerobic threshold (HR_AT_), heart rate at the peak of exercise (HR_max_), pulmonary ventilation at the anaerobic threshold (VE_AT_), pulmonary ventilation at the peak of exercise (VE_max_), and respiratory exchange ratio (RQ) at the peak of exercise.

### Statistics

All statistical analyses were performed using the SPSS 11.0 software (Statistical Package for Social Sciences; SPSS Inc., Chicago, IL, USA). The normal Gaussian distribution of the data was verified by the Shapiro-Wilk test. The Student’s t-test or Mann-Whitney test were employed as appropriate, depending on the distribution of variables. Relationships of interest were assessed by the bivariate correlation analysis. A p-value <0.05 was considered as statistically significant.

## Results

**Fig 1:**
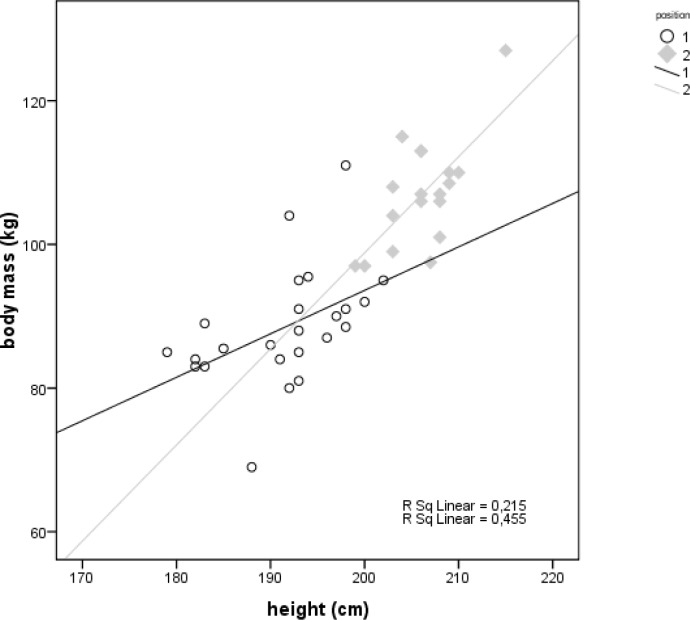
The relationship of body mass and height in male basketball players. Hollow points (1)=perimeter, filled rhombs (2)=post players. Black and gray fit lines represent consecutively perimeter and post players.

**Figure 2:**
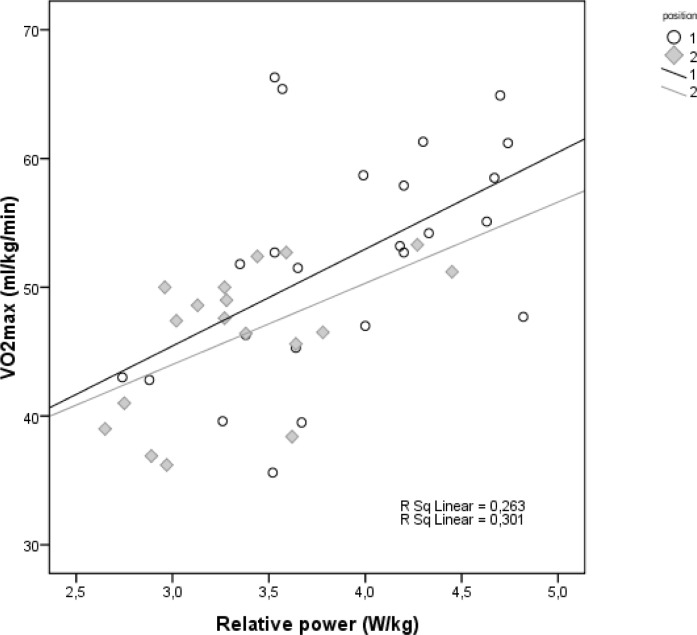
The relationship of oxygen uptake and relative power in male basketball players. Hollow points (1)=perimeter, filled rhombs (2)=post players. Black and gray fit lines represent consecutively perimeter and post players.

Descriptive statistics for the 24 perimeter and 18 post basketball players are shown in Table 1. Student’s t-tests showed statistically significant between-group differences in body height (t=−8.727, p<0.001) and body mass (t=−7.504, p<0.001). Linear regressions of these anthropometric variables are presented in Figure 1. Exercise testing was performed without any adverse effects. The athletes terminated exercise for several reasons, but leg pain and exhaustion were most frequent. The cardiorespiratory test results are presented in Table 2. Shapiro-Wilk tests showed that W_max_, W_AT_, VO_2AT_, RQ, and HR_max_ were non-normally distributed. Mann-Whitney tests of exercise variables between perimeter and post players revealed statistically significant differences in maximal oxygen uptake and relative power, as shown in Table 3. Linear regressions of VO_2max_ and relative power are presented in Figure 2.

## Discussion

This study examined the possible differences in physical work capacity between perimeter and post basketball players. To our knowledge, this is the first attempt to compare the morphological and physiological features of highly trained basketball players using this simplified two-group subdivision rather than the traditional five-position system. The mean indices of cardiopulmonary exercise testing were in the range of those generally reported for highly trained basketball players (Laplaud et al., 2004; Sallet et al., 2005; Cormery et al., 2008; Castagna et al., 2009). As summarized in the comprehensive review of Ziv and Lidor (2009), the VO_2max_ of male basketball athletes is between 50 and 60 ml/kg/min. The majority of athletes in our study showed less developed aerobic properties. It could be associated with mode of testing and phases of the basketball season. The physical effort on a cycle ergometer is associated with 10–20% lower values of VO_2max_ compared to a treadmill, as noted in literature (Arena et al., 2007).

The athletes in our study were tested immediately after the off-season and the obtained values can indirectly reflect some degree of detraining (Marles et al., 2007; Caldwell et al., 2009). Limited aerobic capacity was also shown by Metaxas et al. (2009) in a comparative study of soccer and basketball athletes of distinct levels at the beginning of the pre-season. Also in contrast to the findings of Apostolidis et al. (2004), who reported that the ventilatory threshold occurred in basketball players at 77.6±7.0% VO_2max_, we found that the median VO_2AT_ for perimeter players was 48.15% of VO_2max_, while that of post players was 50.1% of VO_2max_.

The primary findings of this study are as follows: first, the division of players into post and perimeter groups, as frequently done by modern-day coaches based on visual information, has a morphological basis, as shown by our finding of significant between-group differences in body mass and height. These findings are consistent with those of previous studies reporting somatic differences among basketball guards, forwards and centers (Apostolidis et al., 2004; Gocentas et al., 2005; Sallet et al., 2005). Furthermore, we found that our linear regression equations had a differential ability to describe the relationship between body mass and height in the two subgroups. The regression for post players had r^2^=0.45, indicating that this regression can be used as a source of typical values for these players. In contrast, that for the perimeter players had r^2^<0.25, indicating that it should not be used as a source of typical values. In other words, main anthropometric features of perimeter players are highly variable in male basketball. Second, we found statistically significant differences in physical capacity between perimeter and post players. Compared to post players, perimeter players had significantly higher VO_2max_ and relative power. Thus, the division of the players into these subgroups has some physiological basis. Further we found linear relationships between these variables in both subgroups, indicating that these equations can serve as a source of typical values of oxygen uptake and power for male basketball players identified as perimeter or post. These findings are consistent with our previous report of differences in physical capacity among basketball guards, forwards and centers (Gocentas et al., 2005), and with similar results from other studies (Sallet et al., 2005; Cormery et al., 2008).

The present study has two main limitations. First, cycling is not a customary activity for basketball players. Despite this, the benefit of cycle ergometry is related to the direct measure of power output. However, the mechanical efficiency of cycle ergometry was similar for all subjects, arguing in favor of the reliability of this measurement in this study. Second, the presented results should not be taken as direct evidence of the influence of different training regimes on physical capacity in male basketball players. Within the system of performance evaluation criteria (Trninic et al., 2000), it is notable that there are similarities in the criteria for point guards, shooting guards and small forwards, while those for power forwards and centers are similar to one another, but differ from those for the above mentioned positions. Thus, it is likely that players are divided into the perimeter and post subgroups based on similarities in performance criteria, which may result in selection of differences in physical capacity. Nevertheless, the execution of different drills by the subgroups could cause some of the observed between-group differences in physiological features.

The collection of typical or normative physiological variables was recommended for laboratories that collaborate with professional sport teams. Since the number of professional players is limited, not all exercise-testing laboratories are able to collect sufficient data for all five playing positions in basketball. As shown in our study, there are some differences in oxygen uptake and relative power between perimeter and post basketball players. Thus, it may be beneficial for exercise physiologists to consider the two subgroups, rather than the five positions, when collecting data on physical capacity in basketball.

## Figures and Tables

**Table 1:** Basic antropometric characteristics of the investigated athletes

Subgroup	Age (years)	Body height (cm)	Body mass (kg)	BMI
PER	24,13±3,66	191,46±6,31	88,44±8,23	24,14±2,06
POST	25,67±3,22	206,11±3,79	107,00±7,51	25,17±1,31

Subgroup of perimeter players - PER, (n=24); subgroup of post players -POST, n=18).

**Table 2:** Cardiorespiratory test results of elite basketball players divided into perimeter and post subgroup.

Variable	subgroup	min	max	mean	SD	median
W_max_	PER	270	450	343,17	54,394	321
	POST	291	453	356,94	42,651	350,50
W_AT_	PER	101	300	173,5	48,615	157
	POST	123	237	182,44	33,283	196
VO_2AT_	PER	18,1	42,8	25,77	6,244	24,90
	POST	15,6	30,1	23,53	3,667	24,05
VO_2max_	PER	35,6	66,3	52,17	8,661	52,7
	POST	36,2	53,3	46,23	5,576	47,5
VO_2rest_	PER	3,3	6,3	4,8	0,940	4,85
	POST	1,6	6,6	4,76	1,174	4,90
W_relat_	PER	2,74	4,82	3,89	0,590	3,83
	POST	2,65	4,45	3,35	0,485	3,275
VE_AT_	PER	9,8	83,4	51,33	15,085	50,05
	POST	33,4	75,2	57,42	12,288	57,15
VE_max_	PER	93	193,4	136,49	27,444	129,35
	POST	89	199	143,44	25,992	149,6
RQ	PER	1,07	1,32	1,16	0,074	1,135
	POST	1,06	1,41	1,21	0,098	1,19
HR_AT_	PER	101	158	125,63	16,113	124,5
	POST	104	143	123,72	12,256	121,5
HR_max_	PER	141	194	174,25	14,050	177_
	POST	151	190	171,61	12,756	173,5
MET	PER	10,2	18,9	14,87	2,515	15,05
	POST	10,3	15,2	13,21	1,613	13,55

Subgroup of perimeter players - PER, (n=24); subgroup of post players -POST, n=18).

**Table 3:** Non-parametric comparison of maximal oxygen uptake and relative power

	VO_2max_	W_relat_
Mann-Whitney U	125	105
Wilcoxon W	296	276
Z	−2,313	−2,822
P	,021	,005

a Grouping Variable: position (PER/POST)
